# Circulating CD3^+^HLA-DR^+^ Extracellular Vesicles as a Marker for Th1/Tc1-Type Immune Responses

**DOI:** 10.1155/2019/6720819

**Published:** 2019-05-08

**Authors:** Ryutaro Oba, Motomichi Isomura, Akira Igarashi, Kinya Nagata

**Affiliations:** Division of Advanced Technology and Development, BML Inc., Saitama, Japan

## Abstract

Extracellular vesicles (EVs) are known to contain unique proteins that reflect the cells of origins. Activated T cells are reported to secrete various EVs. To establish T cell subset-specific biomarkers, we performed proteomic analysis with Th1- and Th2-derived EVs and identified HLA-DR as a Th1-dominated EV membrane protein. We designed a measurement system for CD3^+^CD4^+^, CD3^+^CD8^+^, and CD3^+^HLA-DR^+^ EVs to specifically determine EV subpopulations derived from CD4^+^, CD8^+^, and Th1-type T cells, respectively. *In vitro* analysis showed that CD3^+^CD4^+^ EVs and CD3^+^CD8^+^ EVs were selectively secreted from activated CD4^+^ and CD8^+^ T cells, respectively, and that CD3^+^HLA-DR^+^ EVs were actively secreted from not only Th1 but also activated CD8^+^ T (probably mostly Tc1) cells. To evaluate the clinical usefulness of these EVs, we measured the serum levels in patients with inflammatory diseases, including Epstein-Barr virus (EBV, *n* = 13) infection, atopic dermatitis (AD, *n* = 10), rheumatoid arthritis (RA, *n* = 20), and osteoarthritis (OA, *n* = 20) and compared the levels with those of healthy adults (*n* = 20). CD3^**+**^CD4^**+**^ EVs were significantly higher in all of EBV infection, AD, RA, and OA while CD3^+^CD8^+^ EVs were higher in EBV infection, lower in RA, and not different in AD and OA relative to the control. The levels of CD3^+^HLA-DR^+^ EVs were markedly higher in EBV infection and significantly lower in AD. The results suggest that these EV subpopulations reflect *in vivo* activation status of total CD4^+^, total CD8^+^, and Th1/Tc1-type T cells, respectively, and may be helpful in T cell-related clinical settings, such as cancer immunotherapy and treatment of chronic infection, autoimmune diseases, and graft-versus-host disease.

## 1. Introduction

Extracellular vesicles (EVs) are 40-2000 nm membranous vesicles secreted by various types of cells and play an essential role in cell-to-cell communication by carrying molecules derived from the cells of origin, including proteins, small nucleic acids, metabolites, and lipids [1–3]. Based on their biogenesis pathways, EVs are often classified into exosomes, microvesicles, and apoptotic bodies [2]. They are secreted from most cell types and released into bodily fluids, including blood, urine, saliva, and breast milk [2], and have attracted attention recently as a new type of biomarkers “liquid biopsy” instead of the conventional invasive measurement techniques [4, 5].

T cells are the principal lymphocytes that play a pivotal role in the immune system. After being sensitized with antigens, T cells display extensive diversity in terms of phenotype and function. Under normal conditions, the majority of T cells are CD4^+^ T helper (Th) 1 and CD8^+^ T cytotoxic (Tc) 1 cells. Both cell types produce type 1 cytokine IFN-*γ* and mediate the major part of type 1 immunity against viral and intracellular bacterial infections and cancer [6, 7]. However, inappropriate activation of Th1/Tc1 cells causes autoimmune diseases and graft-versus-host disease (GVHD) in organ transplantation, while exhaustion and attenuation of these T cell subsets cause infectious diseases and cancer [8–11]. Th2 cells are the principal T cell subset in type 2 immunity which produce type 2 cytokines IL-4, IL-5, and IL-13 and contribute to antihelminth immunity together with mast cells, eosinophils, basophils, etc., while they mediate allergic inflammations such as atopic dermatitis (AD) and asthma and are associated with downmodulation of type 1 immunity [12]. Thus, monitoring of the activation status of particular T cell subsets in the body is important for the treatment of immune-related diseases. Ideally, the monitoring methods should be noninvasive.

So far, various noninvasive methods for monitoring *in vivo* T cell activation have been reported. They include the analysis of peripheral blood T cells on activation/proliferation markers, such as CD25, CD69, and PCNA, and Th1/Th2 phenotypes by flow cytometry (FCM), and measurement of T cell-releasing cytokines and soluble receptors, such as IFN-*γ*, soluble IL-2 receptor, soluble CD4, and soluble CD8, in body fluid samples using ELISA [13–19]. However, peripheral blood T cells do not necessarily reflect local T cell activation, and FCM includes complex procedures. Furthermore, the abovementioned cytokines and soluble receptors are not T cell-specific [20–23]. To our knowledge, a reliable and simple noninvasive method that enables to specifically monitor the activation status of T cells or T cell subsets is currently not available.

In this study, we attempted to establish simple and specific measurement system for the activation status *in vivo* of CD4^+^ T, CD8^+^ T, and Th1-type T cell subsets. For this purpose, we paid attention to T cell-derived EVs because they are released from activated T cells and bear multiple T cell-related molecules, such as T cell receptor, CD3, CD4, and/or CD8, which can in combination define T cell origin of the EVs [24]. Furthermore, a part of T cell-derived EVs is expected to display proteins that characterize Th1-type T cells. Recent progress in cancer immunotherapy has revealed that Th1/Tc1 responses are suppressed in cancer patients and they are restored by blockade of immune checkpoint receptor PD-1 or PD-L1 [25]. Therefore, circulating EVs bearing Th1/Tc1 markers may be useful as predictive and prognostic biomarkers in cancer immunotherapy as well as treatment of infectious and autoimmune diseases.

Previous studies showed that EVs carrying T cell markers CD3, CD4, and CD8 circulate in the blood and are elevated in active chronic hepatitis C and after liver transplantation [26, 27]. In these studies, EVs were detected by FCM and thereby small EVs, such as exosomes, were not included in the assay. EVs bearing CD3, CD4, or CD8a were also detected in normal plasma by using a relatively complicated technology of slide array [28]. Thus, the development of a more convenient and high-throughput technique is desired for the determination of circulating T cell-derived EVs. In the field of cancer, Yoshioka et al. reported a new liquid biopsy technique named ExoScreen to simply and sensitively detect disease-specific circulating EVs using the AlphaLISA system [29]. AlphaLISA is one of the immunoassays and is convenient and high-throughput in that there is no need for washing and the 96-1536 well plate can be used [30]. This technique is considered to be one of the best methods to measure circulating EVs, especially exosome [29]. Thus, in this study, we took advantage of the high sensitivity and convenience of the AlphaLISA system to measure T cell-derived EVs. Yoshioka et al. used dual antibodies to an EV-specific antigen and a cancer-related antigen in the ExoScreen, while we intended to use antibodies to general T cell markers and subset- or activation-specific markers.

Proteomic analysis of EVs has been reported on various immune cells, such as T cells, B cells, macrophages, and dendritic cells [31–35]. However, proteomic studies on EVs derived from T cell subsets such as Th1, Th2, and Th17 are currently limited. In this study, we performed proteomic analysis of EVs derived from human Th1 and Th2 cells and identified HLA-DR as a Th1-dominated EV protein. We then established a high-throughput measurement system for T cell-derived EVs CD3^+^CD4^+^ EVs, CD3^+^CD8^+^ EVs, and CD3^+^HLA-DR^+^ EVs, using the AlphaLISA system and analyzed serum samples obtained from normal adults and patients with various inflammatory diseases. The results suggest that these EVs are useful as serum markers of *in vivo* activation of total CD4^+^ T, total CD8^+^ T, and Th1/Tc1-type T cells, respectively.

## 2. Materials and Methods

### 2.1. Blood Samples

Serum samples from patients with Epstein-Barr virus (EBV) infection, rheumatoid arthritis (RA), or osteoarthritis (OA) were purchased from SUNFCO (Tokyo, Japan). Sera from patients with atopic dermatitis (AD) were purchased from Eolas Biosciences Co. (Chiba, Japan). All donors provided written consent for blood collection and the use of the blood and access to medical records for research purposes. The blood sample collection protocol was approved by the ethics committee of the relevant medical institution. Patients with EBV infection were categorized into primary and recurrent infection based on the serum antibody pattern against EBV-related antigens. All RA patients were positive for the rheumatoid factor and anticyclic citrullinated peptide antibody test, and AD cases were diagnosed as “moderate” or “severe” by registered dermatologists.

Serum and peripheral blood mononuclear cell (PBMC) samples from healthy controls were obtained from volunteers at BML Inc. (Saitama, Japan). All volunteers provided written informed consent, and subject anonymity was preserved using methods approved by the internal Ethics Committee in accordance with the Declaration of Helsinki.

### 2.2. Reagents

The following reagents were used for cell culture: The medium used was RPMI-1640 (Invitrogen, Carlsbad, CA) supplemented with 200 mM L-glutamine, 100 *μ*g/ml streptomycin (Meiji Seika Pharma Co., Tokyo), 100 units/ml penicillin (Meiji Seika Pharma Co.), and 10% EV-free fetal calf serum (FCS). For the preparation of EV-free FCS and EV-free normal human serum (NHS), FCS (Sigma-Aldrich, St. Louis, MO) and mixture of sera from healthy controls were ultracentrifuged at 150,000 ×*g* for 16 hr and the upper half was collected. IL-2 was obtained from Shionogi & Co. (Osaka, Japan). IL-12, IL-4, anti-IL-4 (clone 3007), and anti-IL-12 (clone 24910) were obtained from R&D Systems (Minneapolis, MN). For EV purification, we used the Hybridoma-SFM medium (Thermo Fisher Scientific, Waltham, MA) containing 1–2% EV-free FCS.

The following reagents were used for flow cytometric analysis: Permeabilizing Solution 2, PE-labeled anti-CD3 (clone HIT3a), anti-CD4 (clone SK3), anti-CD63 (clone H5C6), FITC-labeled anti IFN-*γ*/PE-labeled anti IL-4 (clone 25723.11/3010.211), and control mouse IgG2a (clone eBM2a) or mouse IgG1 (clone MOPC-21) were obtained from Becton-Dickinson Biosciences (San Jose, CA). PE-labeled anti-HLA-DR (clone MEM-12) was obtained from Abcam (Cambridge, UK). The following reagents were used for fluorescent nanoparticle tracking analysis: Qdot655 labeled anti-CD3 (clone 4.1) and control mouse IgG2a were obtained from Invitrogen. The following reagents were used for AlphaLISA immunoassay: Biotin-labeled anti-HLA-DR (clone L243), anti-CD63 (clone H5C6), anti-CD4 (clone RPA-T4), and anti-CD8 (clone RPA-T8) were obtained from BioLegend (San Diego, CA).

### 2.3. Th1 and Th2 Cultures

PBMCs were isolated from heparinized peripheral blood using Ficoll Paque (GE Healthcare, Little Chalfont, Buckinghamshire, UK). Naive CD4^+^ T cells were sorted from PBMCs by negative selection using anti-CD45RO- and anti-CD14-coated microbeads (Miltenyi Biotec GmbH, Bergisch Gladbach, Germany) followed by positive selection with anti-CD4-coated microbeads (Miltenyi Biotec). The purity of CD4^+^ cells in the sorted populations was 90-98%. The naive CD4^+^ T cells were stimulated with anti-CD3/CD28-coated beads (Thermo Fisher Scientific) under Th1-inducing condition (100 ng/ml IL-12 and 10 *μ*g/ml anti-IL-4) or stimulated with immobilized anti-CD3 (clone OKT3, BioLegend, 5 *μ*g/ml) and free anti-CD28 (Pharmingen, San Diego, CA, 1 *μ*g/ml) under Th2-inducing condition (100 ng/ml IL-4, 10 *μ*g/ml anti-IL-12, and 10 *μ*g/ml anti-IFN-*γ*). Three days after stimulation, 200 U/ml IL-2 was added to the cultures, and cells were expanded for 2 to 3 weeks in the presence of IL-2. The Th1/Th2 phenotype was examined by intracellular staining of IFN-*γ* and IL-4 as described previously [36]. For purification of EVs, established Th1 and Th2 were secondarily stimulated with immortalized anti-CD3 and free anti-CD28 in the presence of IL-2 (100 U/ml) and IL-12 (5 ng/ml) for Th1 or IL-4 (10 ng/mL) for Th2. After 3- to 4-day culture, the supernatants were collected and subjected to EV purification. For EV purification, the Hybridoma-SFM medium containing 1–2% EV-free FCS was used.

### 2.4. CD4^+^ and CD8^+^ T Cell Cultures

CD4^+^ and CD8^+^ T cells were isolated from PBMCs of healthy volunteers by negative selection with anti-CD56- and anti-CD14-coated microbeads (Miltenyi Biotec) followed by positive selection with anti-CD4- or anti-CD8-coated microbeads (Miltenyi Biotec). The purities of CD4^+^ and CD8^+^ cells in the sorted populations were 90-98%. The obtained CD4^+^ and CD8^+^ T cells were cultured in a medium containing 200 U/ml IL-2 with or without anti-CD3/CD28-coated beads. For purification of EVs, the Hybridoma-SFM medium containing 1–2% EV-free FCS was used.

### 2.5. Purification of EVs

EVs were purified from the culture supernatants using the procedure described in previous studies for the purification of exosome, with some modifications [33, 37]. Briefly, Th1, Th2, CD4^+^ T, or CD8^+^ T cells were cultured in the Hybridoma-SFM medium containing 1–2% EV-free FCS, 200 U/ml of IL-2, and other respective reagents. After 3- to 4-day culture, the cells were removed by centrifugation sequentially twice for 10 min at 2,300 ×*g*. The supernatants were collected and centrifuged for 30 min at 10,000 ×*g*. From the supernatants, EVs were precipitated by centrifugation at 142,700 ×*g* for 2–3 hr at 4°C on a 30% sucrose/PBS/D_2_O cushion. Here, PBS/D_2_O means PBS prepared with deuterium oxide (D_2_O) instead of H_2_O. The cushion was collected and diluted with a large volume of PBS, and EVs were precipitated by the same centrifugation condition (PBS wash). This PBS wash was repeated twice. Finally, the EV-containing cushion was diluted with a large volume of PBS, layered over a sucrose gradient prepared with PBS/D_2_O (0-60% sucrose), and centrifuged for 16 hr at 142,700 ×*g* at 4°C to remove contaminants. The fractions containing CD3^+^CD63^+^ EVs which were determined by AlphaLISA (see below) were collected, and sucrose was removed by ultracentrifugation at 142,700 ×*g* for 2–3 hr at 4°C. The EV pellet was dissolved in a small volume of PBS, and the total protein concentration was determined by a bicinchoninic acid assay (Thermo Fisher Scientific). The purified EVs were stored at −80°C until use. In some experiments, we purified EVs from human sera using a commercial EV isolation kit for serum (Thermo Fisher Scientific) and resuspended the purified EVs to the original volume with EV-free NHS according to the protocol provided by the manufacturer.

### 2.6. Western Blot Analysis

EV samples were dissolved in SDS sample buffer containing 2-mercaptoethanol. After boiling for 10 min, the proteins were separated in 5-20% polyacrylamide gradient gel (ATTO, Tokyo) and then electrically transferred to Immobilon-P nylon membrane (Millipore, Bedford, MA). CD3 and CD4 proteins were visualized using 5 *μ*g/ml of mouse monoclonal antibodies against CD3 (clone: 4C1, Abnova, Taipei, Taiwan) and CD4 (clone:1F6, Bio-Rad, Hercules, CA) and horseradish peroxidase-labeled goat anti-mouse IgG (Zymed Laboratories, San Francisco, CA) followed by chemiluminescent detection using a Western blot chemiluminescence reagent (New England Nuclear, Boston, MA).

### 2.7. Fluorescent Nanoparticle Tracking Analysis

For the analysis of CD3-positive EVs, fluorescent nanoparticle tracking analysis was performed using NanoSight (Malvern Panalytical, Malvern, UK) using the method described in detail previously, with some modifications [38]. Briefly, 3 *μ*l of 900 *μ*g/ml EVs was stained with a final concentration of 0.04 nM of Qdot655-labeled anti-CD3 antibody or control mIgG2a (Invitrogen). After 30 min incubation, the labeled EVs were diluted 500-fold with 0.22 *μ*m filtered PBS and analyzed with NanoSight.

### 2.8. 2D-DIGE and Mass Spectrometry

Two-dimensional fluorescence difference gel electrophoresis (2D-DIGE) and mass spectrometry (MS) analysis were performed for the determination of proteins of interest as described previously, with minor modifications [39, 40]. Briefly, IC5-Osu and IC3-Osu (Dojindo, Kumamoto, Japan) were used for protein labeling according to the protocol supplied by the manufacturer. Fifty *μ*g of purified EVs derived from Th1 and Th2 cells was separately labeled with IC5-Osu (blue) and IC3-Osu (red), in 100 *μ*l of 10 mM HEPES NaOH (pH 8.6). The reaction was stopped by adding 2 *μ*l of 10 mM ethanolamine-HCl, and the labeled samples were washed using a 2D-Clearn up kit (GE Healthcare) and dissolved in 50 *μ*l of 2D-rehydration buffer according to the protocol provided by the manufacturer. Both samples were mixed together and separated by 2D-DIGE performed as described below. For the first dimension, 24 cm nonlinear (pH 3–10) immobilized pH gradient strips (Bio-Lyte 3/10, Bio-Rad) were used with loading 50 *μ*g of each protein sample. Immobilized pH gradient strips were actively rehydrated with the sample at 500 V for 5 hr, and then isoelectric focusing was performed by a linear increase to 3500 V for 1.3 hr and maintained at 3500 V for 17.5 hr with an isoelectric focusing system (Bio-Rad) after adding 1% dithiothreitol. For the second dimension, the immobilized pH gradient strips were equilibrated for 20 min in 50 mM Tris-HCl (pH 8.8), 6 M urea, 30% glycerol, 2% SDS, 1% dithiothreitol, and bromophenol blue and then for 20 min in 50 mM Tris-HCl (pH 8.8), 6 M urea, 30% glycerol, 2% SDS, 2% iodoacetamide, and bromophenol blue. After cutting both ends of the strips, the strips were embedded in 2% (*w*/*v*) agarose on top of 5-20% Realgel plate 1 (Biocraft, Tokyo). The second dimension SDS-PAGE was performed essentially according to the method of Laemmli [41]. The separated labeled proteins in the SDS-PAGE gel were visualized using the Typhoon variable mode imager (GE), and the images were analyzed using ImageQuant Software (Amersham Biosciences). Protein spots were excised from the gels, digested with trypsin and subjected to nano-LC/MS/MS analysis; QSTAR XL (Applied Biosystems, Waltham, MA) and Bio NanoLC (KYA TECH, Tokyo) were used, by a standard protocol that was entrusted to Japan Proteomics (Sendai, Japan).

### 2.9. Flow Cytometric Analysis

To assess the expression levels of various markers on Th1 and Th2 cells, cells were divided into several tubes and stained, in parallel, with PE-labeled antibodies. The stained cells were analyzed on a flow cytometer FACSVerse using BD FACSuite software (Becton-Dickinson Biosciences).

### 2.10. AlphaLISA Immunoassay

An anti-CD3 mAb (clone OKT3, BioLegend) was conjugated to AlphaLISA acceptor beads (Perkin Elmer Inc., Waltham, MA) using the protocol recommended by the manufacturer. Samples were treated by gentle sonication (120 seconds × 3, 100% power, Bransonic; Branson, Danbury, CT) before applying to AlphaLISA assay to disperse EVs. In the AlphaLISA assay, 5 *μ*l of sonicated samples was plated into 4 wells of a 96-well half-area white plate (Perkin Elmer) and 10 *μ*l of reaction buffer (see below) containing 1 mg/ml normal mouse IgG and 100 *μ*g/ml unlabeled OKT3 (for background counts, *n* = 2) or control mouse IgG2a (for sample counts, *n* = 2) was added to 2 wells of the 4 wells. Normal mouse IgG was purified from normal mouse serum (KAC, Kyoto, Japan) with protein G (GE Healthcare). The mixtures were incubated in the dark for 1 hr at room temperature with continuous agitation. Without a washing step, 10 *μ*l of a mixture of 0.5 *μ*g/ml biotinylated mAbs and 50 *μ*g/ml anti-CD3 mAb-conjugated AlphaLISA acceptor beads was added to each well. The mixtures were incubated in the dark for 90 min at room temperature with continuous agitation. Without a washing step, 25 *μ*l of 30 *μ*g/ml AlphaScreen streptavidin-coated donor beads (Perkin Elmer) was added to all wells. All reagents used were diluted in PBS containing 100 mM Tris-HCl (pH 7.5), 1% BSA, 0.4% carboxymethyl cellulose F-120 (Nichirin Chemical, Hyogo, Japan), 2% cold fish gelatin (Sigma-Aldrich), 0.02% proclin 300 (Sigma-Aldrich), 0.1% n-octyl-*β*-D-glucoside (Dojindo), hereinafter referred to as reaction buffer. The mixtures were incubated in the dark for 30 min at room temperature with continuous agitation, and the plate was then read on the EnSpire Alpha 2300 Multilabel Plate reader using an excitation wavelength of 680 nm and emission detection set at 615 nm. The net AlphaLISA counts were obtained by subtracting the background counts from the sample counts, and the average was reported.

### 2.11. Statistical Analysis

The Mann–Whitney *U* test was used for statistical comparisons between two data sets. Differences were considered significant at *P* < 0.05 for all comparisons. The correlation coefficient (*r*) between data sets was calculated by Pearson's correlation coefficient. All statistical analyses were performed using GraphPad Prism software 5.1 for Windows (GraphPad Software, San Diego, CA) or Excel (Microsoft, Redmond, WA).

## 3. Results

### 3.1. Identification of Th1-Derived EV-Dominant Proteins

Th1 and Th2 cells were generated from naive CD4^+^ T cells of healthy volunteers under Th1- and Th2-inducing conditions and expanded over 2 to 3 weeks in the presence of IL-2. The establishment of Th1/Th2 phenotype was determined by intracellular staining for IFN-*γ* and IL-4. The established Th1/Th2 pairs from several healthy donors were secondarily stimulated under Th1- or Th2-inducing conditions, and the culture supernatants at days 3 to 4 were subjected to EV purification as described in Materials and Methods.

**Figure 1:**
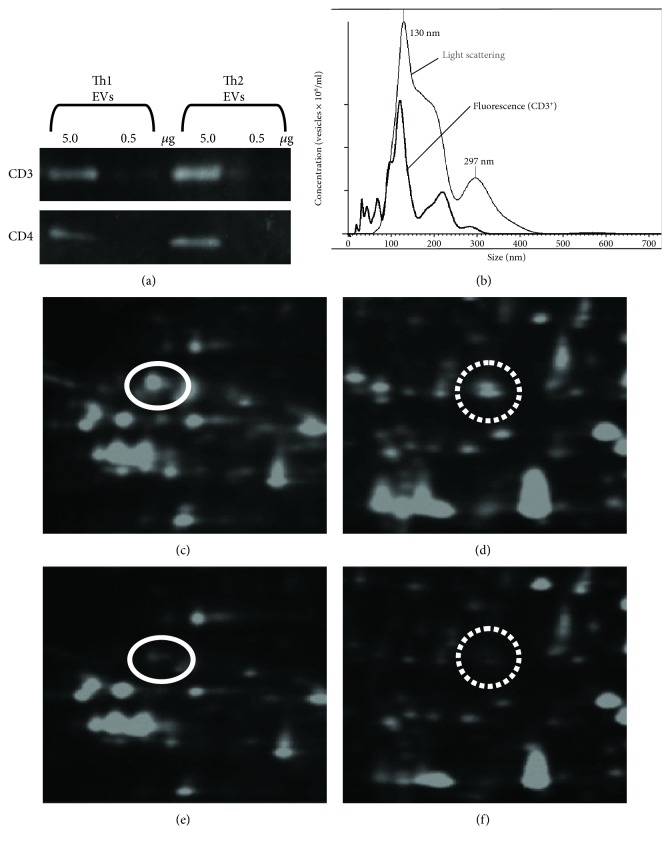
Identification of Th1-derived EV-dominant proteins. (a) Chemiluminescence images. Th1- and Th2-derived EVs were subjected to western blot analysis with anti-CD3 and anti-CD4 mAbs. (b) Fluorescent nanoparticle tracking image. Th1-derived EVs were stained with Qdot655-conjugated anti-CD3 antibody and analyzed on the NanoSight. Results in light scatter (black line) and fluorescence (grey line) modes. (c, d) Two parts of 2D-DIGE images of Th1-derived EV proteins. (e, f) 2D-DIGE images of Th2-derived EV proteins in the same areas as c and d, respectively. Protein spots that showed differences in the fluorescence intensities were picked up for MS analysis. The protein spots indicated by circles (panels c and e, mw = 37067 and pI = 4.8) and dotted circles (panels d and f, mw = 31322 and pI = 7.8) were identified as alpha- and beta-chains of HLA-DR, respectively. Data from one out of six pairs of EVs are shown. The results were similar for the five out of six pairs.

In the next step, we used western blot analysis to evaluate the quality of the purified Th1- and Th2-derived EVs. The results showed that CD3 and CD4 proteins were included in both of Th1- and Th2-derived EVs at nearly equal amounts (Figure 1(a)). Fluorescent nanotracking analysis showed that up to one-third of the purified Th1-derived EVs holds CD3 molecules on their surface (Figure 1(b)). The proportion of CD3-positive EVs in the purified Th2-derived EVs was similar (data not shown).

To identify proteins that could discriminate between Th1- and Th2-derived EVs, several pairs of Th1- and Th2-derived EVs were subjected to comparison of component proteins by 2D-DIGE/MS analysis. Dozens of protein spots that showed differences in the fluorescence intensities between Th1- and Th2-derived EVs were picked up for MS analysis as candidates of the discriminating proteins. In this study, we focused on membrane-associated proteins and found that alpha and beta chains of HLA-DR [42] were dominant in Th1-derived EVs as compared with Th2-derived EVs (Figures 1(c)–1(f)).

### 3.2. Comparison of Various Marker Proteins between Th1 and Th2 Cells

**Figure 2:**
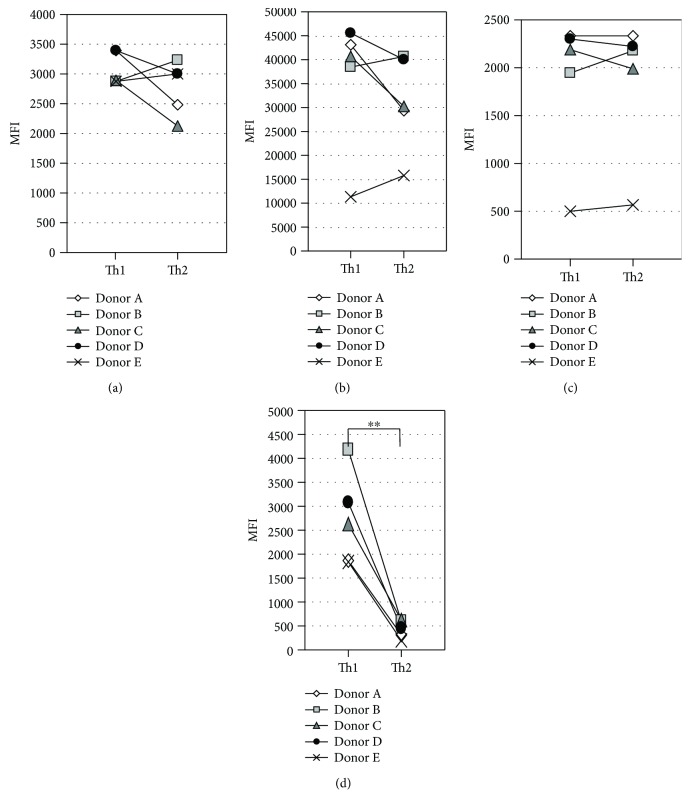
Comparison of various marker proteins between Th1 and Th2 cells. Five pairs of established Th1 and Th2 cells were restimulated with mAbs to CD3 and CD28 under Th1- and Th2-inducing conditions, respectively. After three-day culture, the expression levels of CD3 (a), CD4 (b), CD63 (c), and HLA-DR (d) on the cell surface were measured by FCM. ^∗∗^*P* < 0.01, by the Mann–Whitney *U* test between Th1 and Th2.

HLA-DR is well known as an activation marker of T cells [43], and previous studies reported no significant difference in HLA-DR expression levels between Th1 and Th2 cells [44, 45]. Therefore, we examined HLA-DR expression levels in Th1 and Th2 cells under our culture conditions. The established Th1 and Th2 cells were secondarily stimulated with CD3 and CD28 antibodies under Th1- and Th2-inducing conditions, respectively, and CD3, CD4, CD63, and HLA-DR expression level on the cell surface was measured by FCM at 3 days after stimulation. CD63 is one of tetraspanin family proteins, which are known to be expressed on the EV surface [46]. As shown in Figure 2, no significant difference was evident in the expression levels of CD3, CD4, and CD63 between Th1 and Th2 pairs, while HLA-DR expression level was significantly higher in Th1 than in Th2.

### 3.3. Construction of AlphaLISA to Determine T Cell Subset-Specific EVs

**Figure 3:**
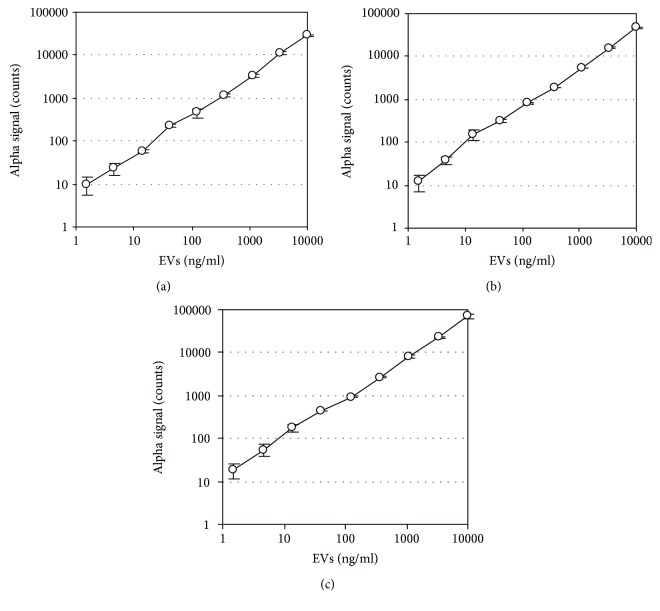
Correlation between concentrations of EVs and AlphaLISA signals. Purified EVs derived from CD4^+^ T cells (a), CD8^+^ T cells (b), and Th1 (c) were subjected to AlphaLISA assay for CD3^+^CD4^+^ EVs, CD3^+^CD8^+^ EVs, and CD3^+^HLA-DR^+^ EVs, respectively. Protein concentrations of EVs were determined with the bicinchoninic acid assay. The correlation coefficient (*r*) between data sets of CD3^+^CD4^+^ EVs, CD3^+^CD8^+^ EVs, and CD3^+^HLA-DR^+^ EVs were 1.000, 0.999, and 0.999, respectively.

To determine the specific EV subpopulations that are derived from CD4^+^ T, CD8^+^ T, common T, and Th1-type T cells, we designed measurement systems for CD3^+^CD4^+^ EVs, CD3^+^CD8^+^ EVs, CD3^+^CD63^+^ EVs, and CD3^+^HLA-DR^+^ EVs, by using AlphaLISA technology. These systems utilized anti-CD3 mAb for capture and mAbs to CD4, CD8, CD63, or HLA-DR for detection. The streptavidin-coated donor beads were excited with a laser at 680 nm, resulting in the release of singlet oxygen, which excited an amplified fluorescent signal in the acceptor bead that emits at 615 nm when the beads were within 200 nm of the captured analyte [47]. During the design of the assay system and following extensive screening of commercial antibodies, we selected several potentially suitable antibodies. In addition, we added relatively high concentration of normal mouse IgG to the reaction buffer to reduce nonspecific reaction and used “net AlphaLISA counts” for measurement to increase the accuracy of the assay. We also found that the addition of certain gelling agents to the reaction buffer significantly increased the assay sensitivity (see Materials and Methods). These improvements allowed easy detection of T cell-derived EVs in normal sera without prior purification or concentration (see below). The established AlphaLISA system showed a linear relationship between alpha signal counts and EVs within a range of concentrations (1.5-10000 ng/ml) (Figures 3(a)–3(c)).

### 3.4. Comparison of T Cell-Derived EVs between Th1 and Th2 Cultures

**Figure 4:**
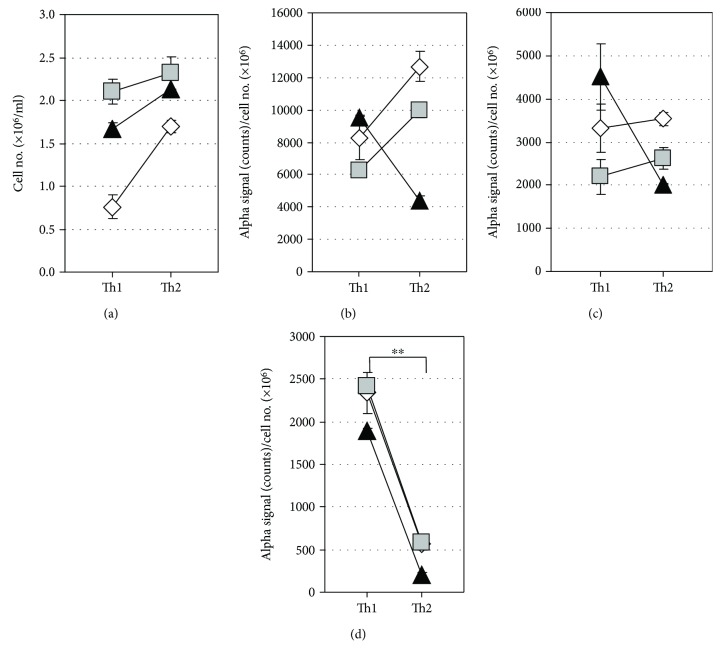
Comparison of T cell-derived EVs between Th1 and Th2 cultures. Three pairs of established Th1 and Th2 cells were restimulated with CD3 and CD28 antibodies under Th1- and Th2-inducing conditions with a starting cell density of 1.0 × 10^6^ cells/ml. After 5-day culture, the cell density was determined by the trypan blue dye-exclusion test (a) and levels of CD3^+^CD4^+^ EVs (b), CD3^+^CD63^+^ EVs (c), and CD3^+^HLA-DR^+^ EVs (d) in the culture supernatants were measured by AlphaLISA. AlphaLISA counts were normalized by dividing the value by the cell number. Data are presented as the mean ± SD. ^∗∗^*P* < 0.01, by the Mann–Whitney *U* test between Th1 and Th2.

Three pairs of established Th1 and Th2 cells were secondarily stimulated with CD3 and CD28 mAbs under the Th1- and Th2-inducing conditions. After 5 days of culture, the supernatants were subjected for the measurement of CD3^+^CD4^+^, CD3^+^CD63^+^, and CD3^+^HLA-DR^+^ EVs by AlphaLISA. Although there was no significant difference in the levels of CD3^+^CD4^+^ and CD3^+^CD63^+^ EVs between Th1 and Th2 cells, the levels of CD3^+^HLA-DR^+^ EVs were significantly higher in Th1 than in Th2 cells (Figure 4(d)).

### 3.5. Levels of T Cell-Derived EVs in CD4^+^ and CD8^+^ T Cell Culture Supernatants

**Figure 5:**
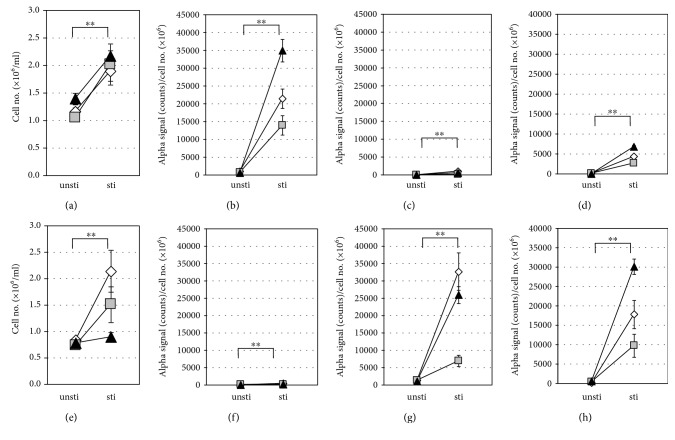
Levels of T cell-derived EVs in culture supernatants of CD4^+^ and CD8^+^ T cells. CD4^+^ and CD8^+^ T cells were purified from PBMCs obtained from three healthy donors and cultured with (sti) or without (unsti) stimulation by CD3 and CD28 antibodies with a starting cell density of 1.0 × 10^6^ cells/mL. After 6-day culture, the cell numbers (a, e) and supernatant levels of CD3^+^CD4^+^ EVs (b, f), CD3^+^CD8^+^ EVs (c, g), and CD3^+^HLA-DR^+^ EVs (d, h) were examined for CD4^+^ T cells (a-d) and CD8^+^ T cells (e-h). AlphaLISA counts were normalized by dividing the value by the cell number. Data are presented as the mean±SD. ^∗^*P* < 0.05 and ^∗∗^*P* < 0.01, by the Mann–Whitney *U* test between sti and unsti cell groups.

Previous studies reported a difference in regulation of HLA-DR expression between circulating CD4^+^ and CD8^+^ T cells in normal and pathological settings [48, 49]. To examine the levels of secreted CD3^+^CD4^+^, CD3^+^CD8^+^, and CD3^+^HLA-DR^+^ EVs by resting and activated CD4^+^ and CD8^+^ T cells, the two types of cells were purified from PBMCs of healthy donors and cultured with and without stimulation by CD3 and CD28 antibodies. After 6 days of the culture, the cell numbers of both CD4^+^ and CD8^+^ T cells were significantly higher in the stimulated cultures relative to the unstimulated cultures (Figures 5(a) and 5(e)). CD3^+^CD4^+^ EVs and CD3^+^CD8^+^ EVs were specifically detected in the stimulated CD4^+^ T and CD8^+^ T cell supernatants, respectively, while secretion of CD3^+^HLA-DR^+^ EVs was detected in both of stimulated CD4^+^ and CD8^+^ T cells (Figures 5(b)–5(d) and 5(f)–5(h)). Interestingly, the levels of secreted CD3^+^HLA-DR^+^ EVs were significantly higher in CD8^+^ T cells than in CD4^+^ T cells (*P* < 0.01). Low EV secretion levels were detected in all unstimulated (resting) T cells (Figures 5(b)–5(d) and 5(f)–5(h)). These results suggested that CD3^+^HLA-DR^+^ EVs are actively released from not only Th1 cells but also Tc1 cells.

### 3.6. Correlation between the AlphaLISA Signal and EVs in Serum Samples

**Figure 6:**
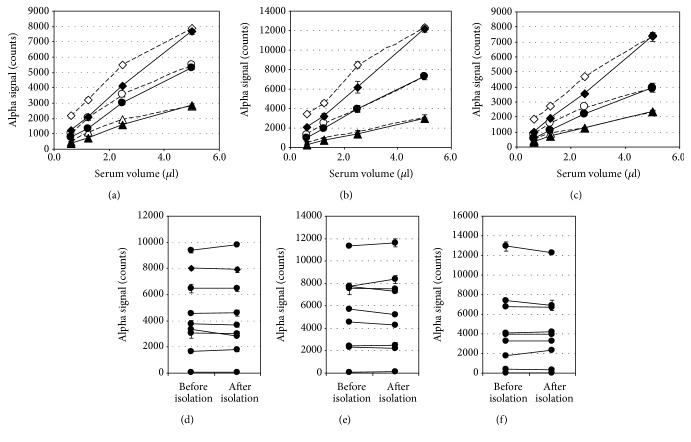
Correlation between the signal and EVs. Three sera from patients with EBV infection (◆◇, ●○, ▲△)were diluted with EV-free NHS (solid lines) or reaction buffer (dotted lines) and subjected to AlphaLISA assay for CD3^+^CD4^+^ EVs (a), CD3^+^CD8^+^ EVs (b), and CD3^+^HLA-DR^+^ EVs (c). The correlation coefficients (*r*) between alpha signal and serum volume in CD3^+^CD4^+^ EV, CD3^+^CD8^+^ EV, and CD3^+^HLA-DR^+^ EV assay ranged from 0.997 to 1.000 when diluted with EV-free NHS, while they ranged from 0.976 to 0.999 when diluted with reaction buffer. EVs were isolated from nine human sera using a commercial isolation kit, suspended to their original volume with EV-free NHS, and their CD3^+^CD4^+^ EVs (d), CD3^+^CD8^+^ EVs (e), and CD3^+^HLA-DR+ EVs (f) were measured together with original sera. The correlation coefficients (*r*) of nine sera between before and after isolation were 0.997, 0.996, and 0.998 for CD3^+^CD4^+^ EVs, CD3^+^CD8^+^ EVs, and CD3^+^HLA-DR^+^ EVs, respectively. Data are presented as the mean ± SD (*n* = 2).

In the next step, we proceeded to the determination of T cell-derived EVs in clinical samples. We first confirmed the correlation between the AlphaLISA signal and serum volume. As shown in Figures 6(a)–6(c), a linear relationship was observed between the alpha signal count and serum volume. The correlation coefficient (*r*) ranged from 0.997 to 1.000 when serum was diluted with EV-free NHS, while it ranged from 0.976 to 0.999 when diluted with reaction buffer. Next, to confirm that the AlphaLISA signals were EV-dependent, we isolated EVs from each serum sample using a commercial isolation kit which has been reported to have better recovery than ultracentrifugation [50], diluted the isolated EVs to the original volume with EV-free NHS, and measured together with original sera. The results showed that the alpha signal count in each serum was almost completely recovered into its EV fraction, supporting the idea that the alpha signals in sera were EV-dependent (Figures 6(d)–6(f)).

### 3.7. Levels of T Cell-Derived EVs in Sera from Patients with Various Inflammatory Diseases

Finally, we evaluated the usefulness of T cell subset-specific EVs in disease settings. For this purpose, we measured CD3^+^HLA-DR^+^, CD3^+^CD4^+^, and CD3^+^CD8^+^ EV levels in sera from patients with EBV infection, AD, RA, and OA, and they were compared with those from healthy subjects. Previous studies showed that EBV infection induces strong Th1/Tc1-type responses while AD is considered to be a typical Th2-type inflammatory disease with upregulated Th2 and downregulated Th1 responses [51, 52]. On the other hand, the skewing to Th1- or Th2-type responses is not distinct in RA and OA. The pathogenesis of RA involves an imbalance of Th17 and Treg, and OA is considered a “wear and tear” disease with inflammatory features [53, 54].

**Figure 7:**
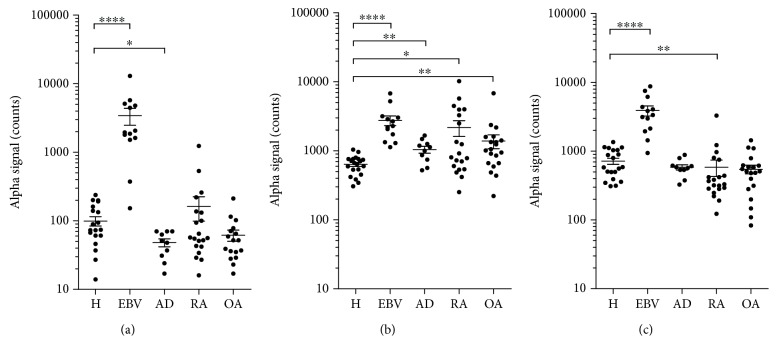
Levels of T cell-derived EVs in sera from patients with various inflammatory diseases. Levels of CD3^+^HLA-DR^+^ EVs (a), CD3^+^CD4^+^ EVs (b), and CD3^+^CD8^+^ EVs (c) in sera from patients with EBV infection (EBV, *n* = 12), atopic dermatitis (AD, *n* = 10), rheumatoid arthritis (RA, *n* = 20), and osteoarthritis (OA, *n* = 20) were measured by AlphaLISA and compared with those of healthy subjects (H, *n* = 20). The bars represent the mean ± SD^∗^*P* < 0.05, ^∗∗^*P* < 0.01, and ^∗∗∗∗^*P* < 0.0001, by the Mann–Whitney *U* test.

As shown in Figure 7(a), serum levels of CD3^+^HLA-DR^+^ EVs were very low in healthy adults, while they were markedly higher in patients with EBV infection and, conversely, significantly lower in patients with AD as compared to healthy adults. On the other hand, the levels of CD3^+^HLA-DR^+^ EVs were not different in RA and OA relative to healthy adults. These results are in accordance with the Th1/Tc1 and Th2 backgrounds of EBV infection and AD, respectively, and support the notion that circulating CD3^+^HLA-DR^+^ EVs reflect Th1/Tc1 status *in vivo*.

The AlphaLISA assay also showed that the levels of serum CD3^+^CD4^+^ EVs were significantly higher in all four disease groups than those of healthy subjects, corresponding to inflammatory feature of these four diseases (Figure 7(b)). On the other hand, the serum levels of CD3^+^CD8^+^ EVs were significantly higher in patients with EBV infection, lower in RA patients, and not different in AD and OA patients relative to the control (Figure 7(c)), according to clinical findings of increased and decreased CD8^+^ T cell counts in EBV infection and RA, respectively [51, 55, 56]. These results support the idea that circulating CD3^+^CD4^+^ and CD3^+^CD8^+^ EVs reflect the activation status of CD4^+^ and CD8^+^ T cells, respectively, *in vivo*.

## 4. Discussion

In this study, we designed a simple and sensitive system for the measurement of T cell-derived CD3^+^CD4^+^ EVs, CD3^+^CD8^+^ EVs, and CD3^+^HLA-DR^+^ EVs. The analysis of culture supernatants from T cell subpopulations and serum samples from patients with various inflammatory diseases suggests that these T cell-derived EVs may be useful as markers for CD4^+^ T cell-, CD8^+^ T cell-, and Th1/Tc1-associated immune responses.

It is reported that the AlphaLISA system emits fluorescent signal when the distance between streptavidin-coated donor beads and acceptor beads is within 200 nm [47]. This notion does not necessarily mean that detected particles have a diameter of 200 nm or less. When two corresponding antigen molecules are located within a distance of 200 nm or less on the particle surface, they will emit fluorescent signal. In our *in vitro* T cell cultures, induction of apoptosis by anti-Fas antibody markedly increased the AlphaLISA signals in the culture supernatants and most of the increased components were eliminated with 0.22 *μ*m filtering (data not shown). Therefore, our ALphaLISA system seems to detect not only exosome and small size microvesicles but also large size microvesicles and apoptotic bodies with particle size exceeding 200 nm.

HLA-DR is composed of alpha and beta chains encoded by the major histocompatibility complex (MHC) class II genes and responsible for the regulation of the immune system like other HLAs [42]. Although the function of HLA-DR in T cells remains uncertain, presentation of class II restricted antigens by T cells has been implicated in energy induction [57]. HLA-DR is also well known as a marker of T cell activation [43]. In this study, we found that HLA-DR is preferentially expressed on the surface of Th1 than on that of Th2 cells. In contrast to this observation, previous studies showed that the surface expression levels of HLA-DR were not significantly different between Th1 and Th2 cells [44, 45]. This discrepancy might be due to the difference in culture and cellular conditions. In previous studies, primary stimulated cultures of CD4^+^ T cells were used for examination, while we used secondary stimulated cultures of once established Th1 and Th2 cells. It is conceivable that regulation mechanisms of HLA-DR expression might differ between differentiating and established Th1/Th2 cells. Our results are in agreement with those of a previous study which showed that MHC class II gene was preferentially expressed in Th1 cells in an IFN-*γ*-dependent autocrine fashion [58]. In addition to T cells, IFN-*γ*-induced expression of HLA-DR was demonstrated in macrophages and epithelial cells [59]. These findings suggest that although HLA-DR expression by itself is not intrinsic to the Th1 phenotype, IFN-*γ*, which was secreted by an autocrine manner, enhanced HLA-DR expression on Th1 cells in our culture conditions. We believe that IFN-*γ*-rich conditions, such as Th1/Tc1 immune responses, enhance HLA-DR expression in T cells in the inflamed sites, which leads to the increase in CD3^+^HLA-DR^+^ EV levels in the circulation.

EVs contain host cell-derived molecules and are attracting attention as “liquid biopsies” [4, 5]. In the present *in vitro* study, we confirmed that CD3^+^CD4^+^ EVs and CD3^+^CD8^+^ EVs were specifically secreted from stimulated CD4^+^ T and CD8^+^ T cells, respectively. Our results also confirmed that CD3^+^HLA-DR^+^ EVs were predominantly secreted from not only stimulated Th1 cells but also stimulated CD8^+^ T cells. The majority of the latter cells were considered to be IFN-*γ*-producing Tc1 cells. Based on these data, we postulate that these T cell-derived EVs may be useful for monitoring the activation status of total CD4^+^ T, total CD8^+^ T, and Th1/Tc1-type T cells *in vivo*, respectively.

According to the above postulation, serum levels of CD3^+^HLA-DR^+^ EVs were markedly elevated in EBV infection, a typical Th1/Tc1-dominated disease, significantly decreased in AD, a Th2-dominated disease, and within the normal range in a lower inflammatory arthritis OA [51, 52]. Although not showing a significant difference, a relatively high mean value of CD3^+^HLA-DR^+^ EVs in the RA group might reflect patients with higher frequency of circulating HLA-DR^+^ T cells [60].

Previous studies of RA lesions demonstrated that the dominant lymphocytes were CD4^+^ T cells, which outnumber CD8^+^ T cells, and that the absolute number of circulating CD8^+^ T cells was within the normal range in the active phase but was significantly lower during the remission period [55, 56, 61]. These reported findings are consistent with our present data that sera from at least some RA patients showed increased CD3^+^CD4^+^ EVs and decreased CD3^+^CD8^+^ EVs levels (Figures 7(b) and 7(c)). Also, in the present study, a significant increase in the CD3^+^CD4^+^ EVs levels was noted in patients with OA. These results are in agreement with previous reports that showed enhanced levels of soluble CD4 in the sera and synovial fluid and significantly higher ratio of circulating CD4^+^ T cells to CD8^+^ T cells in OA than healthy controls [62, 63].

## 5. Conclusion

Although the sample number used in this study was small, we consider that quantification of circulating EVs that are specific to particular T cell subsets is useful for monitoring the activation status of corresponding T cell subsets. Such a strategy may help the treatment of T cell-based diseases, such as cancer, infection, autoimmune disease, and GVHD in organ transplantation.

## Data Availability

Data used to support the findings of this study are available upon request.
